# Association between time of delivery and poor perinatal outcomes -An evaluation of deliveries in a tertiary hospital, South-east Nigeria

**DOI:** 10.1371/journal.pone.0217943

**Published:** 2019-06-10

**Authors:** Paul Eze, Lucky Osaheni Lawani, Chukwuemeka Ikechi Ukaegbe, Okechukwu Bonaventure Anozie, Chukwuemeka Anthony Iyoke

**Affiliations:** 1 Medicins Sans Frontieres (MSF), OCBA, Barcelona, Spain; 2 Department of Obstetrics & Gynecology, Federal Teaching Hospital, Abakaliki, Ebonyi State, Nigeria; 3 Department of Obstetrics & Gynecology, University of Nigeria Teaching Hospital, Enugu, Enugu State, Nigeria; Aga Khan University, PAKISTAN

## Abstract

**Objectives:**

Nigeria account for a significant proportion of adverse perinatal outcome. Nigerian studies assessing impact of time of delivery on perinatal outcome are scarce. This study evaluates any associations between time of delivery and perinatal outcome.

**Methods:**

This was a cross-sectional study at the Federal Teaching Hospital, Abakaliki from 01 January 2016 to 30 June 2018. Data were analysed with IBM SPSS version 25.0.

**Results:**

A total of 4,556 deliveries were analysed. Majority (72.2%) delivered on week days and 27.8% on weekends. Over 90% had 1^st^ and 5^th^ minutes Apgar scores ≥7. There was statistical difference in NICU admission between morning and evening hours (p = 0.009) but not between morning and night hours (p = 0.795). ENND during evening was twice higher (1.2%) than morning (0.5%); p = 0.047 and night hours (0.6%); p = 0.623.There was no difference in the risk of fresh stillbirths between morning and evening (p = 0.560), as well as morning and night hours (p = 0.75), there was also no difference in fresh stillbirths between week days and weekends (p = 0.895). There was no difference in low Apgar scores at 1^st^ minute between morning and evening (p = 0.053) and night (p = 0.221), and between weekdays and weekends (p = 0.524). Similarly, there was no difference in low 5^th^ minute Apgar scores between morning and evening (p = 0.165) and night (p = 0.944), as well as between week days and weekends (p = 0.529). However, ENND was twice (p = 0.085) and 1.3 times higher (p = 0.526) for evening and night hours respectively, while there was no difference between weekends and week days (p = 0.652).

**Conclusion:**

NICU admission and ENND were commoner during evening hours. However, work hours did not affect the rate of stillbirth and low Apgar scores during weekdays and weekends. It is pertinent for each obstetric unit to identify and modify factors responsible for unfavourable outcomes during various shifts, with the aim of improving perinatal health.

## Introduction

Consistently delivering optimal care irrespective of the time or day of delivery is critical to sustaining the maternal and neonatal health gains achieved in the last decade, especially in resource-poor developing countries [1, 2]. Often times, there are unsubstantiated and non-evidence-based opinions among health care providers on whether labour and its outcome are associated with poorer results at night and over weekends, when hospital services may not be at their optimal best. While some scholars have reported that childbirth and its complication do not differentiate between hours of the day nor days of the week, others have reported contrary findings [3, 4]. The time of delivery may be regarded as an indirect indicator of organizational vulnerability, as conditions may be more suboptimal during evening and night or during weekend [5]. There are reports that increased fatigue, limited access to senior obstetric support and reduced out-of-office hours resources are factors that could impact the quality of care particularly night shifts and to some extent weekends [3], as there may be fewer and less experienced doctors available on duty at nights and during the weekends [4].

Recent studies on the variation of time and day of delivery on perinatal outcomes have reported varying or inconclusive results. While some studies have demonstrated increased adverse outcomes for deliveries during night shifts [2, 3,5–12] and weekends [7, 13–16], some other studies have reported no increased in adverse effects for babies born during night shifts [1, 4, 17, 18] or weekends [4, 10, 19–22]. However, it is important to note that these studies applied different time definitions in their analysis. Although over 90% of neonatal deaths occur in sub-Saharan Africa, most of which are due to preventable intra partum factors [23], most of the recent studies that assessed the impact of time and day of delivery on perinatal outcomes were ironically performed in high income countries [8, 10, 11, 14–22]. Their findings though technically valid cannot be entirely representative of events in resource-poor settings due to significant differences in the level of health care advancement.

A recent study in Tanzania evaluated the perinatal outcomes of 2,636 hospital deliveries and reported that fresh stillbirths, neonatal distress and neonatal deaths were more significantly associated with deliveries occurring during night shifts compared to evening and morning shifts [1]. However, this study included preterm babies in their analysis of perinatal outcomes. Given that preterm delivery is an established significant risk factor for still births and low or abnormal Apgar scores [24], we aim to exclude such significant confounder in our evaluation of perinatal outcomes by assessing deliveries of term babies born at different hours of the day and days of the week. If significant morbidity and/or mortality are demonstrated during such shifts, this would suggest modification of identified factors which can be addressed to improve outcomes [4].

Therefore, using data from a tertiary health institution in Southeast Nigeria, we seek to know if there are differences in the perinatal outcomes; live birth, fresh stillbirth, Apgar scores, admission into the neonatal intensive care unit (NICU) and early neonatal death (ENND) of term babies delivered at different hours of the day and days of the week. This study, hopefully, will provide quality evidence that could help improve decision making in obstetric units of most resource-poor settings.

## Materials & methods

This was a cross-sectional study of all the deliveries at the Federal Teaching Hospital, Abakaliki (FETHA) between 01 January, 2016 and 30 June, 2018. Ethical approval was given by the Ethics Review Board of the Federal Teaching Hospital, Abakaliki.

The Federal Teaching Hospital, Abakaliki (FETHA) is a tertiary hospital in Abakaliki, the capital of Ebonyi state in Southeast Nigeria. The obstetrics unit has an annual delivery rate of about 2,400 births and offers 24hrs services. The unit comprises of five teams, made up of consultant Obstetricians and resident Doctors.

One of the five teams is responsible for the labour/delivery room from 8:00 am to 3:59 pm during weekdays (Mondays to Fridays). The team on call duty takes over responsibility of the labour/delivery room from 4:00 pm until 7.59 am the next day. On each day of weekends (Saturdays and Sundays), a different team on call takes responsibility for the labour/delivery room for 24 hours. Each team is made up of 3–4 Obstetricians, 5–8 resident doctors and 4 house officers. The length of working hours for nurses was 6 hours for morning shift (8:00 am to 1:59 pm), 4 hours for evening shift (2:00 pm to 5:59 pm) and 14 hours for night shift (6:00 pm to 7:59 am). The nurses run the same three shifts all days of the week, Mondays to Sundays. There are usually 4 nurses on morning shift, 3 nurses on afternoon shift and 4 nurses for night shift.

All high-risk deliveries are conducted by senior resident doctors and/or consultant Obstetricians; and attended by a Neonatologist. The standard practice in the hospital is to admit all neonates with an abnormal Apgar score of <7 into the Neonatal Intensive Care Unit (NICU). Irrespective of their Apgar score at birth, sick neonates are also admitted into the NICU.

All the mothers who delivered in FETHA during the study period were identified from the delivery register and assessed for eligibility for inclusion or exclusion from the study. Written consents were obtained from all parturient who delivered during the study period for their medical records to be used for research purposes when necessary. The age range of the participants was 19–42 years.

The data of parturient were made fully anonymous before they were accessed. Data were collected using a pre-tested specially-designed pro forma. Data on socio demographic characteristics were extracted from the case notes. Other information extracted were the time of delivery, the APGAR scores (1^st^ minute and 5^th^ minute), admission into NICU and ENND and birth weight.

Timing of birth variables were defined as:

1. Hour of birth: Morning hours (08:00 am to 3:59 pm), Evening hours (04:00 pm to 11:59 pm), or Night hours (12:00 am to 07:59 am)
2. Day of the week: Weekday (Monday to Friday) versus Weekend (Saturday or Sunday).

Data were analyzed with IBM SPSS version 25.0. Analysis involved descriptive and inferential statistics. Student’s t-test (2-tailed, unequal sample size) was used for continuous variables, and Pearson’s chi-squared (2-tailed, unequal sample size) for categorical variables. Multiple Logistics analysis were performed to determine the adjusted odds ratio (AOR) for the effect of work hours (morning hours, evening hours, night hours and weekends) on adverse perinatal outcomes. P<0.05 was used to define statistical significance at 95% confidence interval (CI).

## Results

**Fig 1:**
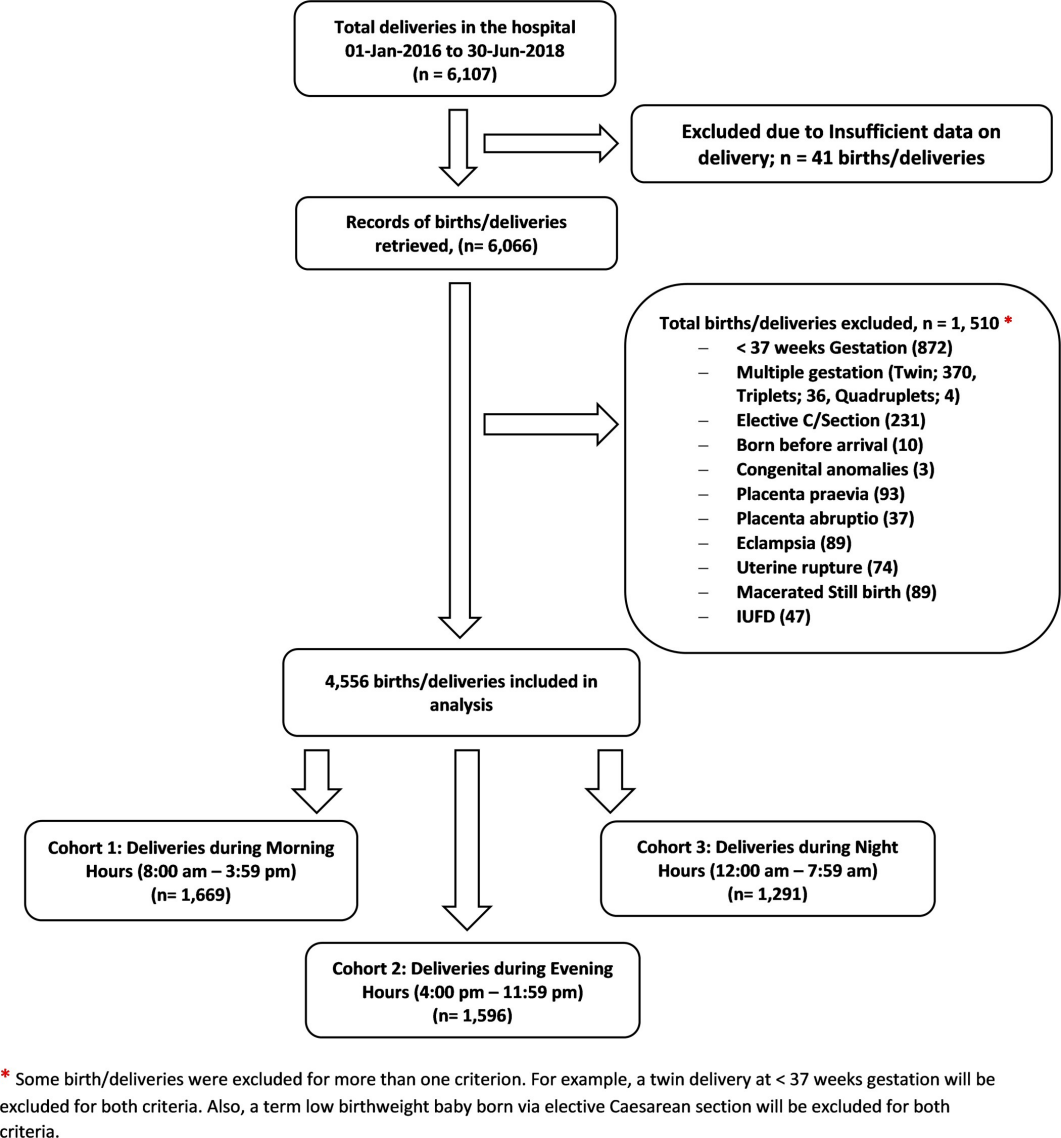
Flowchart for selection of study cohorts.

A total of 4,556 deliveries met the inclusion criteria and were also eligible for analysis. Fig 1: Flowchart for Selection of Study Cohorts depicts the total deliveries, selection of subjects and the numbers of subjects in each cohort. Table 1 shows that over 4/5^th^ of parturient managed during the three different work hours was aged 20–34 years. Over 2/5^th^ of participants in the three cohorts had secondary level of education. Most subjects (>80%) received at least one antenatal care from a skilled birth attendant. Over 4/5th of the participants had vaginal delivery, while the remainder had caesarean delivery (p = 0.449). The parity, gestational age at delivery and other socio-demographic characteristics are shown in Tables 1 and 2.

**Table 1:** Socio-demographic characteristics of Study cohorts by time of delivery (morning, evening and night hours).

Socio-demographic characteristics		Morning Hours ^#^ (8:00 am– 3:59 pm) N = 1,669 n (%)	Evening Hours (4:00 pm– 11:59 pm) N = 1,596 n (%)	*P*-value	Night Hours (12:00 am– 7:59 am) N = 1,291 n (%)	*P*-value
**Age of the Parturient mothers**	< 20 years	34 (2.0%)	44 (2.8%)	0.303	32 (2.5%)	0.685
	20–34 years	1513 (90.7%)	1425 (89.2%)		1161 (89.9%)	
	≥ 35 years	122 (7.3%)	127 (8.0%)		98 (7.6%)	
**Marital status**	Married	1642 (98.4%)	1562 (97.9%)	0.303	1273 (98.6%)	0.653
	Single	27 (1.6%)	34 (2.1%)		18 (1.4%)	
**Educational status**	None	90 (5.4%)	95 (6.0%)	0.088	71 (5.5%)	0.130
	Primary	365 (21.9%)	298 (18.7%)		251 (19.4%)	
	Secondary	703 (42.1%)	696 (43.6%)		563 (43.6%)	
	Post-secondary	511 (30.6%)	507 (31.8%)		406 (31.4%)	
**Religion**	Christian	1646 (98.6%)	1570 (98.4%)	0.568	1271 (98.5%)	0.758
	Muslim and others	23 (1.4%)	26 (1.6%)		20 (1.5%)	
**Received at least 1 antenatal care**	Received antenatal care	1430 (85.7%)	1337 (83.8%)	0.131	1119 (86.7%)	0.453
	No antenatal care	239 (14.3%)	259 (16.2%)		172 (13.3%)	
**Parity**	0	474 (28.4%)				0.703
			502 (31.5%)	0.058	384 (29.7%)	
	1–4	1055 (63.2%)	944 (59.1%)		(62.3%)	
	≥ 5	140 (8.4%)	150 (9.4%)		103 (8.0%)	
**Gestational Age**	Mean (Std Dev) in weeks	39.58 (± 1.285)	39.55 (± 1.278)	0.463	39.54 (± 1.222)	0.340
	37^0^–41^6^ weeks	1619 (97.0%)	1547 (96.9%)	0.919	1257 (97.4%)	0.579
	≥ 42 weeks	50 (3.0%)	49 (3.1%)		34 (2.6%)	
**Mode of Delivery**	Vaginal Delivery	1343 (80.5%)	1254 (78.6%)	0.193	1060 (82.1%)	0.123
	Caesarean Section	326 (19.5%)	342 (21.4%		231 (17.9%)	

^#^ Day hours (8:00 am– 3:59 pm) is the reference group.

**Table 2:** Comparison of characteristics of study cohorts by day of delivery (Weekday vs Weekend).

Socio-demographic characteristics		Weekdays^#^ (Monday to Friday) N = 3,290 n (%)	Weekends (Saturday & Sunday) N = 1,266 n (%)	*P*-value
**Age of the Parturient mothers**	< 20 years	72 (2.2%)	38 (3.0%)	0.231
	20–34 years	2962 (90.0%)	1137 (89.8%)	
	≥ 35 years	256 (7.8%)	91 (7.2%)	
**Marital status**	Married	3236 (98.4%)	1241 (98.0%)	0.448
	Single	54 (1.6%)	25 (2.0%)	
**Educational status**	None	201 (6.1%)	76 (6.0%)	0.942
	Primary	649 (19.7%)	257 (20.3%)	
	Secondary	1391 (42.3%)	531 (41.9%)	
	Post-secondary	1049 (31.9%)	402 (31.8%)	
**Religion**	Christian	3243 (98.6%)	1244 (98.3)	0.498
	Muslim and others	47 (1.4%)	22 (1.7%)	
**Received at least 1 antenatal care**	Received antenatal care	2822 (85.8%)	1064 (84.0%)	0.148
	No antenatal care	468 (14.2%)	202 (16.0%)	
**Parity**	Median	1.00	1.00	
	0	988 (30.0%)	372 (29.4%)	0.708
	1–4	2013 (61.2%)	(62.4%)	
	≥ 5	289 (8.8%)	104 (8.2%)	
**Gestational Age**	Mean (Std Dev) in weeks	39.56 (± 1.255)	39.54 (± 1.290)	0.570
	37^0^–41^6^ weeks	3200 (97.3%)	1223 (96.6%)	0.239
	≥ 42 weeks	90 (2.7%)	43 (3.4%)	
**Mode of Delivery**	Vaginal Delivery	2653 (80.6%)	1034 (81.7%)	0.449
	Caesarean section	637 (19.4%)	232 (18.3%)	

^#^ Weekdays (Monday to Friday) is the reference group.

Table 3 shows the neonatal anthropometry of the babies delivered during the study period. Majority of babies born weighed 2500-3999grams- 88.9% of them were delivered during morning hour, 88.8% and 90.0% delivered during evening and at night hours respectively. The proportion that weighed <2500g and >4000g are shown in Table 2. More than 95% of participants had live births during the three different work hours- Table 3. The Apgar scores at 1^st^ and 5^th^ minutes for majority (>90%) of participants in the three groups were ≥7. There was statistical significance between the first minute Apgar scores of babies delivered during morning and evening hours (p = 0.013) but not for those delivered during night hours (p = 0.399). There was no difference in 5^th^ minute Apgar scores for all the work hours- Table 3.

**Table 3:** Neonatal anthropometry and perinatal outcomes by time of delivery (morning, evening and night hours).

PERINATAL INDICATORS / DAY OF DELIVERY			Morning Hours ^#^ (8:00 am– 3:59 pm) (n = 1,669)	Evening Hours (4:00 pm– 11:59 pm) (n = 1,596)	*P*-value	Night Hours (12:00 am– 7:59 am) (n = 1,291)	*P*-value
	**Birth weight**	Mean (Std Dev) in gram	3258 (± 465)	3234 (± 481)	0.154	3241 (± 475)	0.333
		< 2500 gram	50 (3.0%)	65 (4.1%)	0.162	52 (4.0%)	0.031*
		2500 to 3999 gram	1484 (88.9%)	1417 (88.8%)		1162 (90.0%)	
		≥ 4000 gram	135 (8.1%)	114 (7.1%)		77 (6.0%)	
	**Head circumference**	Mean (Std Dev) in cm	34.89 (± 2.70)	34.97 (± 2.85)	0.373	34.87 (± 2.89)	0.849
		< 37 cm	1487 (89.1%)	1409 (88.3%)	0.473	1161 (89.9%)	0.470
		≥ 37 cm	182 (10.9%)	187 (11.7%)		130 (10.1%)	
	**Length**	Mean (Std Dev) in cm	48.51 (± 3.07)	48.45 (± 3.04)	0.615	48.41 (± 3.157)	0.375
**Perinatal Outcomes of current delivery**	**Birth outcome**	Live birth	1612 (96.6%)	1529 (95.8%)	0.272	1251 (96.9%)	0.678
		Still birth	57 (3.4%)	67 (4.2%)		40 (3.1%)	
	**APGAR Scores at 1 minute**	Normal (7–10)	1493 (92.6%)	1378 (90.1%)	0.013*	1148 (91.8%)	0.399
		Low/Abnormal (0–6)	119 (7.4%)	151 (9.9%)		103 (8.2%)	
	**APGAR Scores at 5 minutes**	Normal (7–10)	1574 (97.6%)	1476 (96.5%)	0.070	1224 (97.8%)	0.801
		Low/Abnormal (0–6)	38 (2.4%)	53 (3.5%)		27 (2.2%)	
	**Admission into NICU**	Not Admitted	1530 (94.9%)	1417 (92.9%)	0.009*	1191 (95.2%)	0.795
		Admitted post-delivery	82 (5.1%)	112 (7.3%)		60 (4.8%)	
	**Early Neonatal Death**	Death within 7 days of birth	8 (0.5%)	18 (1.2%)	0.047*	8 (0.6%)	0.623

^#^ Morning hours (8:00 am– 3:59 pm) is the reference group.

*Statistically significant

There was statistical difference in NICU admission between morning (82/1669; 5.1%) and evening hours (112/1596; 7.3%); p = 0.009, but not between morning and night hours (p = 0.795). ENND during evening was twice higher (18/1596; 1.2%) than morning (8/1669; 0.5%); p = 0.047 and night (8/1291; 0.6%); p = 0.623 hours- Table 3.

Logistic regression- Table 4, indicates that there was no difference in the risk of fresh still births between morning and evening (AOR = 1.119, CI = 0.766–1.636, p = 0.560), as well as morning and night (AOR = 0.920, CI = 0.599–1.415, p = 0.75) work hours. Also, there was no statistical difference in fresh still births between week days and weekends (AOR = 1.024, CI = 0.715–1,469, p = 0.895). There was no difference in low Apgar scores at 1^st^ minute for evening (AOR = 1.303, [CI- 0.996–1.704]; p = 0.053) and night (AOR = 1.201, [CI = 0.895–1.612]; p = 0.221) hours and between weekdays and weekends (AOR = 1.085, [CI = 0.845–1.393]; p = 0.524). Similarly, for 5^th^ minute Apgar scores for evening (AOR = 1.366, [CI = 0.880–2.120]; p = 0.165) and night (AOR = 0.982, [CI = 0.585–1.647]; p = 0.944) hours, as well as between week days and weekends (AOR = 0.870, [CI = 0.564–1.342]; p = 0.529).

**Table 4:** Adjusted odds ratios (with 95% confidence intervals) for the effect of time and day of delivery on adverse perinatal outcomes.

Variables	Time (Hour) of Delivery (Reference group is Morning Hours)						Day of Delivery (Reference group is Weekday)		
	Evening Hours (4:00 pm– 11:59 pm)			Night Hours (12:00 am– 7:59 am)			Weekend (Saturday & Sunday)		
	AOR	Confidence Interval	*p*-value	AOR	Confidence Interval	*p*-value	AOR	Confidence Interval	*p*-value
Fresh stillbirth (Intra-partum death)	1.119	0.766–1.636	0.560	0.920	0.599–1.415	0.705	1.024	0.715–1.469	0.895
Low/Abnormal Apgar scores at 1 minute	1.303	0.996–1.704	0.053	1.201	0.895–1.612	0.221	1.085	0.845–1.393	0.524
Low/Abnormal Apgar scores at 5 minutes	1.366	0.880–2.120	0.165	0.982	0.585–1.647	0.944	0.870	0.564–1.342	0.529
Admission into the Neonatal Intensive Care Unit (NICU)	1.390	1.017–1.900	0.039*	0.977	0.680–1.402	0.898	1.053	0.782–1.417	0.735
Early Neonatal Death	2.147	0.901–5.116	0.085	1.391	0.501–3.862	0.526	0.833	0.375–1.849	0.653

AOR- Adjusted odds ratio

*Statistically significant

NICU admission was 1.3 times higher for evening hours compared to other work hours (AOR = 1.390, [CI = 1.017–1.900]; P = 0.039). Also, the risk of ENND was twice higher (AOR = 2.147, [CI = 0.901–5.116]; p = 0.085) and 1.3 times higher (AOR = 1.391, CI = 0.501–3.862]; p = 0.526) for evening and night hours respectively, while AOR = 0.833, CI = 0.375–1.847, p = 0.652 for weekends compared to week days.

## Discussion

The current study determined if there were any significant differences in the perinatal outcomes; live birth, fresh stillbirth, Apgar scores, admission into NICU and ENND’s of term babies delivered during different work hours of the day and weekends. Overall, this study showed that NICU admission and ENND were commoner in neonates born during evening work hours. (4:00 pm to 11:59 pm) compared with those born during the morning hours (8:00 am to 3:59 pm). However, work hours did not affect the rate of still birth and Apgar scores during work days and between weekdays and weekends. Similar results were reported in hospitals in the Netherlands and in the United States of America [5, 18, 22, 25].

Most of the participants in the present study were within the reproductive age 20–34 years, which could have limited the obstetrics risk associated with pregnancies at extremes of age, which have been reported to be associated poor perinatal outcomes [2, 26]. Majority of babies born weighed 2500–3999 gram with no disproportionate difference in other neonatal anthropometry. This removes the effect of extremes of weight; such as low birth weight and fetal macrosomia which are possible confounders for poor outcome in this study.

The Apgar scores at 1^st^ and 5^th^ minutes for majority of participants in the three groups were normal (≥7). The first minute Apgar scores of babies delivered were significantly poorer during evening hours compared with morning hours but not for those delivered during night hours. Could this have been due to less optimal nature of hospital services, absence of more experienced care providers or fatigue of attending skilled care providers after normal office hours? Mgaya in Tanzania reported that neonatal distress was more significantly associated with deliveries during night shift compared to morning and evening shifts [2], but this finding was at variance with the report by Suzuki in Japan, where umbilical artery PH was less than 7 during day time shift (8am-4pm) compared to other work hours [9]. In the present study, fifth minute Apgar scores for all work hours was however not significantly different for all cohorts. Also, in the current study, there was no significant difference in both the first and 5^th^ minutes Apgar scores between week days and weekends. Although, some researchers have questioned the use of only Apgar score, which is subjective and have recommended its use in conjunction with acid-base PH and lactate for assessment of immediate neonatal status. Unfortunately, this assessment tool is still not widely available or non-existent in most resource constrained settings like ours where the current study was conducted, thus necessitating the use of Aapgar score alone as the only option or tool available. However, conventional-Apgar scores and combined-Apgar scores are still widely used in the immediate assessment of the status of the new born in most low and high income settings [24, 25].

The present study also showed that NICU admission for neonates delivered during evening work hours was more than morning hours, but not so for night hours compared to morning hours. This finding was similar to the finding by Jensen et al in the United States, where they reported higher neonatal morbidity for low birth weight babies delivered during off peak hours, necessitating NICU admission [27]. These admissions were mainly due to intra-partum events, since most of the high risk patients with obstetric problems were excluded to avoid bias. These adverse intra-partum events could vary with timing of labour and delivery [2, 9, 15].

More than 95% of participants had live births during the three different work hours. Although our study results did not demonstrate any significant statistical difference in live births or fresh stillbirth between deliveries during morning hours and those that occurred during evening and night hours, as well as between week days and weekends, there was increased in early neonatal deaths for deliveries in the evening hours (twice higher) compared to deliveries in the morning hours but not between week days and weekends. Several other studies have report increased adverse perinatal outcomes for deliveries outside the morning hours [2, 3, 5–12], as well as during weekends [15]. ENND may be related to unfavourable events of labour, fetal distress and failed resuscitation which may all be related to timing of birth and prevailing factors during different work hours, as well as perverse organizational vulnerability that overwhelms most hospitals in resource-poor settings leading to suboptimal services [1–3, 5]. In the United States, Salihu et al reported that neonatal mortality rate was higher on weekends (3.25/1000) compared to weekdays (2.87/1000) [15]. The insignificant CI of AOR may have been due to other confounders like institutional challenges of service delivery associated with work hours and clients’ condition or complications which occurred prior to presentation, both of which were difficult to adjust for during logistic regression.

Overall, we found that there were increased adverse perinatal outcomes for deliveries during evening hours compared to deliveries during morning hours, but not for weekdays compared to weekends. This finding was consistent with similar studies in the United States where adverse outcome was higher during off-office hours like night shift [4, 18]. Conversely, results from studies in Ireland [3] and the United Kingdom [17] did not demonstrate any increased adverse perinatal outcome for deliveries outside off-office hours. Regarding outcome between weekdays and weekends, our findings were consistent with reported from Canada [21] and Japan [19], which showed that there was no statistically significant difference in adverse perinatal outcome for deliveries during weekend (Saturday and Sunday) compared to weekdays.

The strength of this study was mainly the availability of complete records and its sample size which gives it a high power. Additionally, data for our study are quite recent reflecting current practice in the institution. However, its limitations are related to some short-comings associated with conventional Apgar score assessment which include inter-ratter variability of Apgar scores and the subjective nature of this scoring system. The study also did not capture information concerning babies who were discharged earlier than seven days and could have died at home, as well as babies whose parents opted to be discharged home against medical advice or to other hospitals for further care. There is need for a larger prospective multi-centre study with longer follow up period and incorporation of other methods of evaluating immediate neonatal outcome such as blood gas and acid-base balance.

## Conclusion

In conclusion, NICU admission and ENND were commoner during evening work hours. However, work hours did not affect the rate of still birth and low Apgar scores during weekdays and weekends. It is pertinent for each obstetric unit to identify and review factors responsible for unfavourable outcomes during various work hours, with the aim of correcting gaps to improve perinatal health outcomes and neonatal health indices.
